# The Oncogene Metadherin Interacts with the Known Splicing Proteins YTHDC1, Sam68 and T-STAR and Plays a Novel Role in Alternative mRNA Splicing

**DOI:** 10.3390/cancers11091233

**Published:** 2019-08-23

**Authors:** Hayley J. Luxton, Benjamin S. Simpson, Ian G. Mills, Nicola R. Brindle, Zeba Ahmed, Vasilis Stavrinides, Susan Heavey, Stefan Stamm, Hayley C. Whitaker

**Affiliations:** 1 Molecular Therapeutics and Diagnostics Group, University College London, London W1W 7TS, UK; 2 Uro-Oncology Research Group, Cancer Research UK Cambridge Institute, University of Cambridge, Cambridge CB2 0RE, UK; 3 Nuffield Department of Surgical Sciences, John Radcliffe Hospital, Oxford OX3 9DU, UK; 4 Institute for Health Sciences, Centre for Cancer Research and Cell Biology, Queens University Belfast, Belfast BT9 7AE, Ireland; 5 Apoptosis and Proliferation Control Laboratory, The Francis Crick Institute, London NW1 1AT, UK; 6 Department of Molecular & Cellular Biochemistry, University of Kentucky, Lexington, KY 40508, USA

**Keywords:** alternative splicing, CD44, YTHDC1, SAM68, prostate cancer

## Abstract

Oncogenic metadherin is a key contributor to tumourigenesis with metadherin expression and cytoplasmic localisation previously linked to poor survival. A number of reports have shown metadherin localises specifically to nuclear speckles known to be rich in RNA-binding proteins including the splicing proteins YTHDC1, Sam68 and T-STAR, that have been shown to select alternative splice sites in mRNA of tumour-associated proteins including BRCA, MDM2 and VEGF. Here we investigate the interaction and relationship between metadherin and the splice factors YTHDC1, T-STAR and Sam68. Using a yeast two-hybrid assay and immunoprecipitation we show that metadherin interacts with YTHDC1, Sam68 and T-STAR and demonstrate that T-STAR is significantly overexpressed in prostate cancer tissue compared to benign prostate tissue. We also demonstrate that metadherin influences splice site selection in a dose-dependent manner in CD44v5-luc minigene reporter assays. Finally, we demonstrate that prostate cancer patients with higher metadherin expression have greater expression of the CD44v5 exon. CD44v5 expression could be used to discriminate patients with poor outcomes following radical prostatectomy. In this work we show for the first time that metadherin interacts with, and modulates, the function of key components of splicing associated with cancer development and progression.

## 1. Introduction

Metadherin, also known as LYRIC/AEG1, plays a pivotal role in cancer and is reported to have many functions and subcellular localisations including mRNA binding in the endoplasmic reticulum (ER). Additionally, some have suggested nuclear roles such as the inhibition of apoptosis via FOXO3a and FOXO1 in endocrine cancers, the regulation of c-myc expression through the transcriptional repressor promyelocytic leukemia zinc finger (PLZF) and the modulation of cell cycle progression through BRCA2-and CDKN1A-interacting protein a (BCCIPa) [1,2,3,4,5,6]. During prostate cancer development, metadherin has been shown to translocate from the nucleus to the cytoplasm, indicating that an important nuclear role of metadherin is lost during prostate tumourigenesis [7,8].

Alternative splicing of pre-mRNA is a major driver of protein diversity and affects more than 95% of multi-exonic coding human genes [9]. Upregulation of endogenous alternatively spliced tumourigenic variants is frequently observed in many cancers such as the proangiogenic splice variant of vascular endothelial growth factor (VEGF_165_), an epithelial-mesenchymal transition-inducing isoform of Rac1 (Rac1b), and the cancer-initiating form of protein kinase C (PKCβII) [10,11,12,13]

YTHDC1 is a ubiquitously expressed nuclear protein involved in splice site selection that localises to YT bodies; dynamic compartments, which first appear at the beginning of S-phase in the cell cycle and disperse during mitosis [14]. These sub-nuclear speckles co-localise with several known spliceosome proteins including SRSF2 [14,15,16]. Using minigene reporter assays YTHDC1 has been shown to modulate mRNA splice site selection in a concentration-dependent manner [17,18]. More recently, the ability of YTHDC1 to splice endogenous transcripts has been demonstrated for vascular endothelial growth factor (VEGF), breast cancer 1 (BRCA1) and the progesterone receptor (PGR) [19,20]. Sam68 is associated with many hallmarks of cancer including epithelial-mesenchymal transition via stabilisation of the splicing factor SF2/ASF, cell cycle progression through Cyclin D1b, inhibition of apoptosis via BCL-X, cell migration through the cell surface protein CD44, and enhanced cell survival in response to DNA damage [21,22,23,24]. Sam68, the related protein T-STAR and YTHDC1 all localise to Sam68 nuclear bodies which traffic mRNAs through the nucleus [25,26,27,28]. 

Here we describe a novel function for nuclear metadherin as a modulator of pre-mRNA processing. We show that metadherin modulates alternative splicing via interactions with known splicing factors YTHDC1, Sam68 and T-STAR, potentially revealing novel tumour biomarkers and therapeutic targets. 

## 2. Results

### 2.1. Metadherin Interacts with Splicing Protein Ythdc1

**Figure 1:**
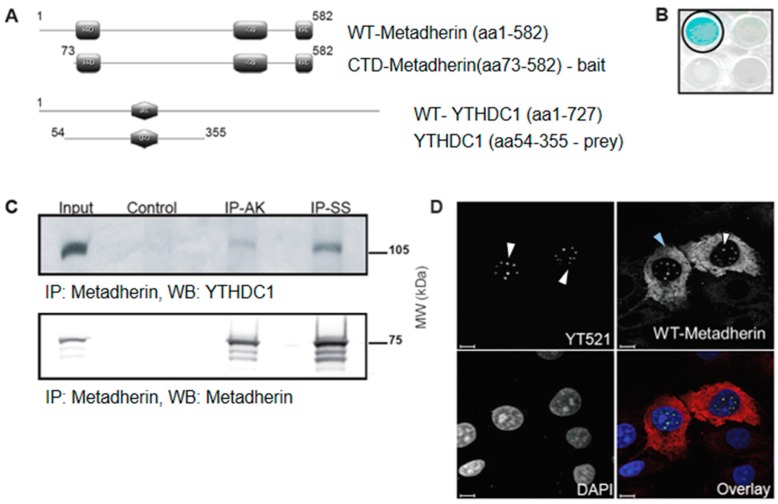
Metadherin interacts with YTHDC1 in the nucleus. (**A**) Schematic representation of metadherin (upper panel) and YTHDC1 constructs (lower panel). Lysine-rich domains (KRD) of metadherin shown in grey rectangles (top), and glutamine rich domain (QRD) of YTHDC1 shown in grey hexagon (bottom). (**B**) Yeast two-hybrid assay using pLexA-DIR-metadherin-aa73–582 (C-terminal domain (CTD)-metadherin) as bait and a human placental cDNA library. β-Galactosidase assay confirms interaction between YTHDC1 and metadherin, YTHDC1 well circled in black, adjacent wells show alternate targets which were all negative. (**C**) Endogenous metadherin was immunoprecipitated using two antibodies against a central epitope (IP-AK) and C-terminal epitope (IP-SS) with metadherin from 1 mg of COS7 protein lysate. Input is 10 µg protein lysate, sheep IgG was used as control. Western blots were probed for YTHDC1 and metadherin (SS). Blots were visualised with enhanced chemiluminescent luminol-based (ECL) substrate plus or, when the signal exceeded the dynamic range of film, using 3,3’-Diaminobenzidine (DAB). (**D**) COS7 cells were fixed in methanol, probed with an anti-YTHDC1 (red) and anti-metadherin (green) antibodies, and mounted with DAPI (blue). White arrows show nuclear localization of the target protein, blue arrows indicate cytoplasmic localization. Images were obtained using a Nikon Eclipse confocal microscope using a ×100 objective. Scale bars represent 10 μm.

A human placental cDNA library and the metadherin C-terminal domain (CTD, amino acids (aa) 73–582) were used in a yeast two-hybrid assay, which identified aa54–355 of YTHDC1 as a metadherin-interacting domain (Figure 1A,B). The interaction between endogenous YTHDC1 and metadherin was confirmed in mammalian cells by immunoprecipitation using two anti-metadherin antibodies: AK (IP-AK) or SS (IP-SS) [29] (Figure 1C).

Metadherin has been associated with several sub-cellular localisations including nuclear speckles associated with FOXO3a and PLZF [4,5]. Using confocal microscopy, we showed that nuclear wt-metadherin co-localised completely with YTHDC1 in sub-nuclear speckles, most likely YT bodies (Figure 1D). When wt-metadherin was over-expressed, YTHDC1 retained its previously described co-localisation with the well-characterised splice factor SRSF2, suggesting that YTHDC1 may associate with spliceosomal proteins and co-localise with metadherin within YT bodies (Figure S1, Supplementary Materials) [14].

### 2.2. Sam68-Associated Protein T-Star is Androgen-Regulated and Overexpressed in Prostate Cancer

**Figure 2:**
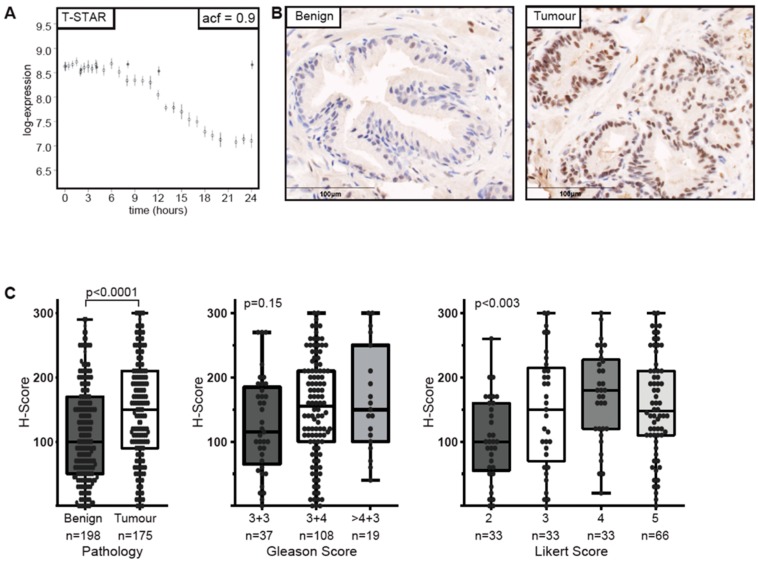
T-STAR is overexpressed in prostate cancer. (**A**) Androgen regulation of T-STAR determined by expression analysis of LNCaP cell lines treated with synthetic androgen (open circles) or vehicle control (filled circles) over 24hrs. ACF = autocorrelation function. (**B**) Examples of immunohistochemistry (IHC) of T-STAR showing benign (left) and tumour (right) tissue. Staining is shown in brown with nuclei shown in blue. (**C**) Staining was analysed by h-score and analysed based on pathology (left), Gleason score (centre) and Likert score (right). Dots represent individual patients, whiskers extend to the minimum and maximum data points, the boxes represent data from first to third quartile, and the horizontal line represents the mean. Significance was analysed using two-tailed Mann–Whitney test.

The known spliceosome protein, Sam68, is reported to interact with the androgen receptor (AR) and modulate the splicing of the receptor itself as well as AR targets [30,31].These reports led us to investigate the androgen regulation of proteins associated with alternative splicing using expression data generated from the LNCaP cell line treated with synthetic androgen (R1881) or vehicle control (EtOH). Results indicated androgen regulation of the Sam68-related protein T-STAR, however, no androgen regulation was observed for probes against metadherin, SAM68 or YTHDC1 (Figure 2A and Figure S2, Supplementary Materials).

The STAR proteins are splicing factors with well characterised roles in spermatogenesis, adipogenesis, neuronal development and tumourigenesis [30,32,33,34]. Using immunohistochemistry we established the over expression of T-STAR in prostate tumourigenesis showing that nuclear T-STAR expression was low in benign tissue and upregulated in tumour tissue based on a two-tailed Mann–Whitney test (*p* < 0.0001) [35] (Figure 2B,C). Interestingly, T-STAR expression also associated with Likert score, an ordinal score (ranging from 1–5) that denotes the likelihood of a significant tumour on magnetic resonance imaging (MRI, Figure 2C), but was not significantly associated with Gleason Grade despite a trend towards higher expression with increasing Gleason Grade (*p* = 0.15). Taken together these results suggest a link between splicing proteins and tumourigenesis.

**Figure 3:**
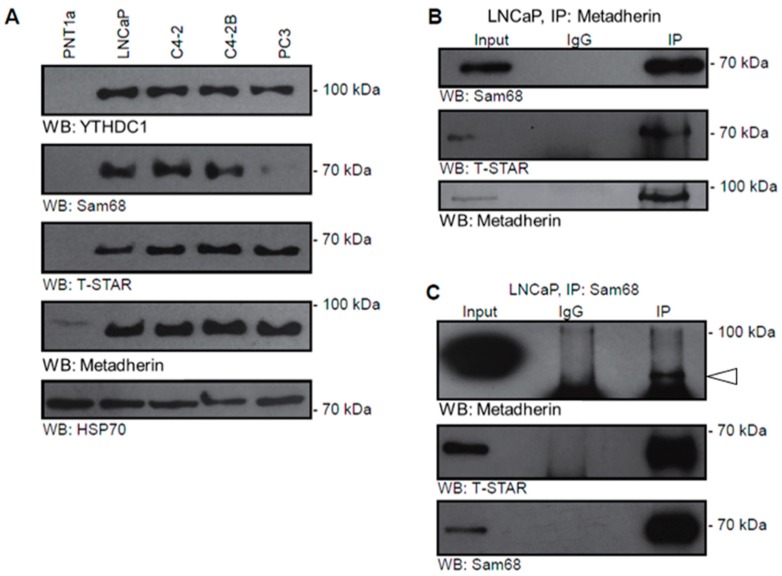
T-STAR interacts with metadherin and is overexpressed in prostate cancer. (**A**) Expression of YTHDC1, Sam68, T-STAR and metadherin were examined in protein lysates from prostate cancer cell lines PNT1a, LNCaP, C4-2, C4-2B and PC3. (**B**) Endogenous metadherin was immunoprecipitated from 1mg LNCaP protein lysate with 10µg protein lysate used as input and rabbit IgG as a negative control. (**C**) Endogenous SAM68 was immunoprecipitated from 1 mg LNCaP protein lysate. 10 µg protein lysate was used as input, rabbit IgG as a negative control. White arrow indicates the band corresponding to metadherin.

As we saw altered expression of T-STAR in prostate cancer, we used a panel of prostate cell lines to examine the protein expression of alternative splicing components, specifically Sam68, T-STAR, YTHDC1 and metadherin (Figure 3A). All of the splice factors examined were detected in LNCaP cells and the LNCaP-derived prostate cancer cell lines C4-2 and C4-2B, but not in the benign prostate cell line PNT1a supporting a role in tumourigenesis. We also failed to detect Sam68 in the androgen independent PC3 cell line.

### 2.3. Metadherin Directly Interacts with Splicing Complex Proteins Sam68 and T-Star

To determine if metadherin interacts with these other splicing complex proteins we immunoprecipitated (IP) metadherin from LNCaP whole cell lysates and detected interactions between metadherin and both Sam68 and T-STAR (Figure 3B). Using an anti-SAM68 antibody, reciprocal interactions could also be detected between T-STAR, metadherin and Sam68 (Figure 3C). The authors noted that the reciprocal IP of the band for metadherin was slightly obscured by the signal from the 50kDa light chain band below therefore was highlighted (Figure 3C). Additionally, the bands detected in IP lane run slightly lower than input lane due to the increased concentration of salts and detergent in the radioimmunoprecipitation assay (RIPA) buffer. These results confirmed the existence of a complex containing metadherin and other, well characterised, splicing proteins and taken with our localisation data it suggests these interactions most likely occur in the nucleus.

### 2.4. Metadherin Attenuates the Effect of Ythdc1 on Cd44v5-Luc Splicing

**Figure 4:**
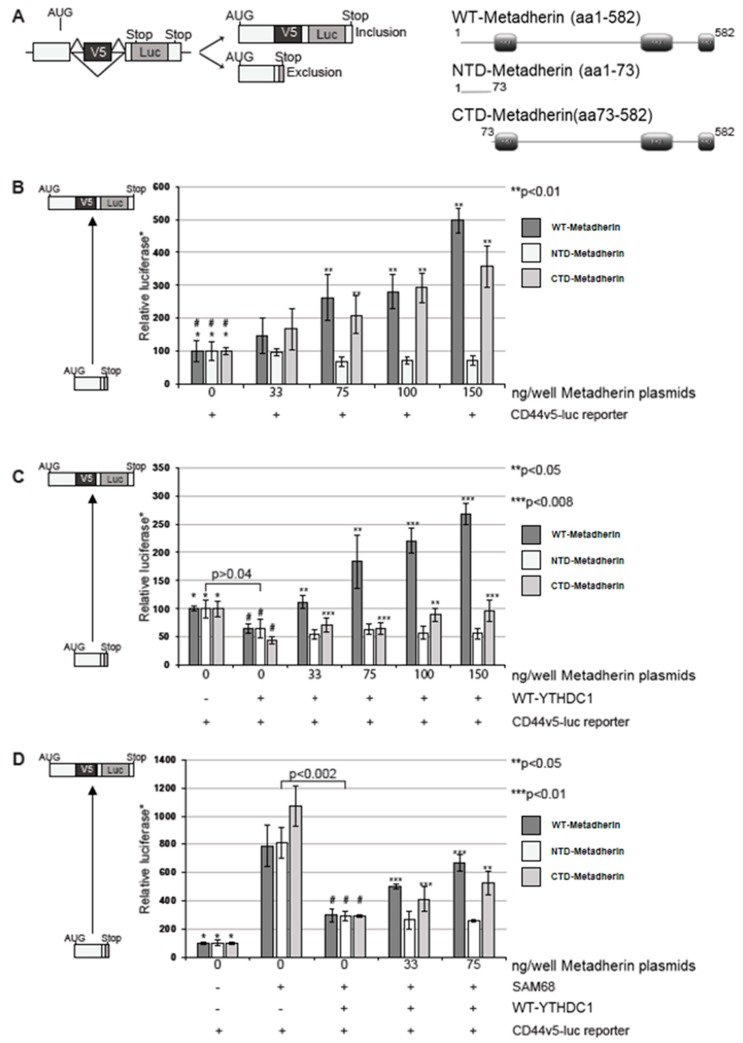
Metadherin promotes exon inclusion during splicing. (**A**) Left: Schematic representation of CD44v5-luc construct with v5 exon inclusion (top) and exclusion (bottom) Right: Schematic representation of metadherin constructs with wild-type (WT)-metadherin (top), N-terminal domain-metadherin (NTD-metadherim) (middle) and C-terminal domain metadherin (CTD-metadherin) (bottom) (**B**). COS7 cells were transfected with CD44v5-luc and increasing concentrations of the metadherin constructs: WT-metadherin (dark grey bars), NTD-metadherin (empty bars), CTD-metadherin (light grey bars). BOS β-galactosidase plasmid was also transfected to control for transfection efficiency. Luciferase activity normalised using a β-galactosidase activity and reported relative to the reporter alone (*). (**C**) COS7 cells were transfected with CD44v5-luc, BOS-β-galactosidase, YTHDC1 and increasing concentrations of the metadherin constructs and assayed as in (B). (**D**) COS7 cells were transfected with CD44v5-luc, BOS-β-galactosidase, YTHDC1, Sam68 and increasing concentrations of the metadherin constructs, grown and assayed as in (B). Schematics left of plots show exon exclusion represented by low relative luciferase and exon inclusion with high relative luciferase. Grey triangle represents increase in exon inclusion. P-values were calculated using a Student’s two-tailed *t*-test. **/*** indicate significant differences compared to bars marked “#”.

As we have shown that metadherin interacts with alternative splicing proteins we investigated if metadherin had a functional effect on splicing by assessing the inclusion of exon v5 in the CD44v5-luc minigene (Figure 4A, left) [5]. Previous reports have shown the larger C-terminal domain of metadherin interacts with the transcriptional repressor PLZF, and the N-terminal domain interacts with the microtubule organising protein BCCIPα so we used both wild-type (wt) and domain constructs of metadherin to assess their ability to influence splicing activity [5,6]. Firstly we examined the ability of metadherin alone on its ability to influence CD44v5-luc minigene splicing. Both the wt- and the C-terminal domain (CTD)-metadherin constructs could significantly activate CD44v5-luc splicing (*p* < 0.01) resulting in an increase in luciferase detection whereas N-terminal domain (NTD)-metadherin tended to reduce exon inclusion (Figure 4B). These data support a role for metadherin in alternative splicing and build upon previous results implicating metadherin in RNA binding in the endoplasmic reticulum [1].

YTHDC1 is known to repress exon inclusion of the CD44v5-luc minigene [17,18]. To assess the impact of YTHDC1 and metadherin together on alternative splicing, the metadherin constructs were overexpressed alongside YTHDC1 in mammalian cells transfected with the CD44v5-luc minigene (Figure 4C). As expected YTHDC1 caused a significant reduction in exon skipping compared to the CD44v5-luc reporter gene alone confirming ability of YTHDC1 to recognise alternative splice sites (*p* < 0.04) (Figure 4B) [17]. The addition of wt-metadherin resulted in an increase in exon inclusion that abolished the exon skipping effect of YTHDC1 (Figure 4C). CTD-metadherin was also able to induce exon inclusion, to levels equivalent to baseline. No effect was observed with NTD-metadherin. This indicates that metadherin may regulate the effect of YTHDC1 in alternative splicing.

Sam68 has previously been shown to increase exon inclusion and as expected, the addition of Sam68 increased CD44v5 exon inclusion in a dose-dependent manner up to 75ng/well (Figure S4A, Supplementary Materials) [21]. As shown by Hartmann et al. YTHDC1 induced exon skipping even in the presence of Sam68 (Figure 4D) [17]. However, the addition of either wt-metadherin or CTD-metadherin resulted in an increase in exon inclusion in a dose-dependent manner (Figure 4D). Once again NTD-metadherin had no effect on the ability of YTHDC1 to exclude the V5 exon suggesting that the C-terminal domain of metadherin, also contained with the wt-metadherin construct is essential for its interaction with YTHDC1 and its role in alternative splicing.

**Figure 5:**
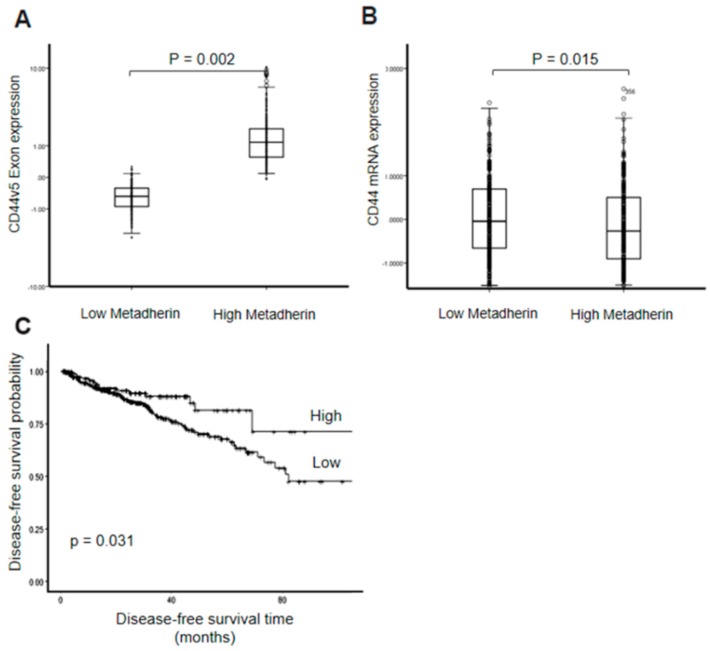
Increased metadherin expression is associated with increased CD44v5 exon expression and altered disease free survival. (**A**) CD44v5 mRNA expression levels between The Cancer Genome Atlas (TCGA) prostate adenocarcinoma (PRAD) patients separated by median metadherin expression. (**B**) Overall CD44 mRNA expression between patients separated by median metadherin expression (**C**) Kaplan-Meier showing disease-free survival time in patients segregated by CD44v5 mRNA expression. *P*-values were calculated using a Student’s two-tailed *t*-test and a log-rank test respectively.

Finally, our evidence suggested a relationship between metadherin and the splicing of CD44 in vitro, however, it remained to be seen if a relationship existed in an endogenous context. To this end, we attempted to further confirm this in a patient cohort. The Cancer Genome Atlas (TCGA) prostate adenocarcinoma (PRAD) cohort was used to compare CD44v5 exon expression between patients with either high or low metadherin expression (separated by median expression). There was a significant increase in the expression levels of CD44v5 mRNA between patients with low and high metadherin expression (*p* = 0.002), concordant with our observation that increased metadherin results in greater inclusion of the CD44v5 exon leading to greater expression (Figure 5A). Another interpretation of this result may be that metadherin and CD44 are co-expressed leading to this pattern of expression. Comparing metadherin mRNA expression to that of full-length CD44 did reveal a weak but significant difference, however, in the opposite direction (*p* = 0.015) indicating that there was a specific effect on the inclusion of exon v5 in tumours with high metadherin expression not explained by simple co-expression (Figure 5B). To investigate the potential clinical significance of this relationship, we compared the disease-free survival of patients with high and low CD44v5 expression (expression was discretized into high and low expressers using the MaxStat R package). The comparison found a significant difference in disease-free survival between low and high expressers (*p* = 0.031) with median disease-free survival for low CD44v5 patients being 82.3 months and the median for high CD44v5 expressers, not reached (Figure 5C).

## 3. Discussion

Metadherin has previously been shown to regulate processes important in cancer such as mRNA binding within the ER, regulation of apoptosis and regulation of c-myc expression [1,2,3,4,5,6]. Here, for the first time, we demonstrated a novel nuclear role for metadherin, where it binds to YTHDC1 and co-localise in sub-nuclear speckles to regulate splicing. Our observation is consistent with previous studies in which, metadherin has been shown to localise to nuclear speckles and to fine-tune many nuclear functions, including the regulation of expression of tumour promotor FOXO3a, the attenuation of promyelocytic leukaemia zinc finger protein (PLZF)-mediated transcriptional repression and the direct modulation of tumour-associated BCCIPα degradation [3,4,5,6]. Additionally, it has been described that nuclear translocalisation of metadherin occurs upon stimulation with TNF-α [36]. However, this is the first time metadherin has been shown to be associated with splicing machinery.

Other splicing proteins such as Sam68 have been demonstrated to interact with the AR which modulates key genes in this process [31]. Androgens play a key part in the development and progression of prostate cancer. Here, we demonstrated that Sam68-associated protein T-STAR expression was dependent on stimulation from synthetic R1881 in LNCaP cells. Moreover, the expression of metadherin and splisosomal proteins YTHDC1, Sam68 and T-STAR were all absent in benign prostate cell-lines and expressed highly in tumourigenic lines further suggesting a link between splicing machinery and tumuorigenesis. To this end, we also examined the expression of T-STAR in prostate cancer biopsy cores and adjacent benign tissue from a total of 249 men from the prostate imaging compared to transperineal ultrasound-guided biopsy for significant prostate cancer risk evaluation (PICTURE) trial. T-STAR expression was significantly upregulated in tumour cores compared to adjacent benign tissue. This observation, while intriguing, somewhat contradicts our study in LNCaP cells showing a strong negative androgen regulation. As our experimental model only looked at R1881 response up to 24 hours, it may be that long-term compensatory mechanisms act to reverse this change. Alternatively, the high variation of T-STAR expression within our cohort may suggest that some patients have undergone regulatory changes in the machinery regulating androgen signalling, although this is purely speculative. T-STAR expression has previously been shown to be altered in breast cancer and correlated with estrogen receptor negativity and HER2 status suggesting alternative signalling pathways regulating T-STAR may well exist [37]. More simply, T-STAR mRNA levels may not correlate well with T-STAR protein expression. Our observation that T-STAR becomes overexpressed in cancer complements work on closely related STAR family member Sam68, shown to interact with T-STAR, which has previously been shown to have oncogenic functions and is associated with poor prognosis in several cancers [22,23,38]. Other splicing factors such as proline- and glutamine-rich/non-POU domain-containing octamer-binding protein (PSF/NONO) and heterogeneous nuclear ribonucleoprotein (HNRNP) have also been shown to become dysregulated in prostate cancer where they become key driving forces for progression [39].

Additionally and unexpectedly, we also found that increased T-STAR expression also significantly associated with higher Likert tumours. Likert score has been demonstrated to correlate with Gleason grade [40]. T-STAR weakly associated with Gleason grade, however, as this association was not significant it may be that T-STAR expression correlates more highly with another unknown tumour feature detected by multi-parametric MRI. This moderate association is noteworthy, as few studies have been carried out to determine which biological processes determine MRI visibility and those that have often describe genes associated with cell growth as well as AR signalling to be upregulated in MRI visible lesions [41,42].

Given our observation that metadherin interacts with splicing protein YTHDC1, we clarified if there was also potential interaction with alternative splicing proteins T-STAR and Sam68. Our results demonstrated that there was a clear interaction between metadherin and all four splicing proteins cementing the assumption that metadherin associates with splicing machinery.

Next, we looked at how metadherin influenced the splicing in an experimental system using a minigene derived from the human CD44 gene. CD44 is highly studied in human malignancy and specific splice-variants of CD44 have been causally related to metastatic behaviour in a variety of carcinomas [43]. CD44 has been suggested as a target for immunotherapy or as a treatment, using antibodies conjugated to anti-tumour agents [43]. We discovered that both WT and CTD-metadherin could significantly activate CD44v5-luc splicing. Furthermore, we found that YTHDC1, previously demonstrated to repress exon inclusion in the same system, could be overridden through the addition of WT-metadherin, suggesting that the interaction between metadherin and YTHDC1 may sequester YTHDC1 away from RNA binding sites, thus reducing any repressive effect. Alternatively, metadherin could repress YTHDC1 exon skipping by recruiting repressor proteins, similar to its previous nuclear role with PLZF [5]. Interestingly, this override could not be achieved through the addition of Sam68 as previously found. These effects were only present with either WT or CTD-metadherin in opposed to NTD-metadherin indicating that the C-terminal domain is crucial for metadherin’s regulation of alternative splicing. 

Finally, we attempted to confirm our observations in a clinical context, finding increased CD44v5 expression in prostate cancer patients who have high metadherin expression. This supported our in vitro assay findings, suggesting increased inclusion of the CD44v5 exon in patients with higher metadherin; this was despite having an overall negative correlation with full-length CD44 mRNA. Furthermore, this relationship may have some clinical significance as reduced CD44v5 expression was associated with reduced disease-free survival following radical prostatectomy. These findings may be of particular relevance in cancer therapy, where metadherin has recently been highlighted as an important therapeutic target [44].

## 4. Materials and Methods 

### 4.1. Cell Culture

All cells were purchased from ATCC. COS-7 cells were routinely cultured in Dulbecco’s modified Eagle’s media (DMEM). PNT1a, LNCaP, C4-2 and C4-2B cells were routinely cultured in Roswell Park Memorial Institute (RPMI) 1640 media. All media was supplemented with 10% fetal bovine serum. 

### 4.2. Plasmids

Wild-type (wt) metadherin(metadherin-aa1-582) and the C-terminal domain (metadherin-aa73-582) were cloned from T7-wt-metadherin [45] into mammalian pSG5 using BamH1 and EcoR1 restriction sites. The N-terminal domain (metadherin-aa1-73) was cloned into the CMV-Myc vector using EcoRI and NotI. CD44v5-minigene plasmid was a kind gift from Harald König (Institute of Toxicology and Genetics, Germany) [46]. YTHDC1-B constructs were kindly provided by Stefan Stamm (University of Kentucky, USA) [17]. Sam68 was a gift from Tetsu Akiyama (University of Tokyo, Japan) [47].

### 4.3. CD44 Minigene Assays

CD44v5-minigene plasmid was transfected into cells alongside wt-metadherin, N-terminal metadherin or C-terminal metadherin and a BOS β-galactosidase plasmid to control for transfection efficiency (25 ng/well) [48]. Empty pSG5 vector was used to equalise the amount of DNA transfected. After 48 hours cells were harvested on ice for 15 minutes using reporter lysis buffer (50 mM Tris HCl pH 7.2, 0.1% Tween-20, 1 mM CaCl_2_, 1 mM MgCl_2_). Luciferase activity was measured using the Luclite luciferase assay kit (Packard Biosciences). Results were normalised using a β-galactosidase activity measured with a Galacton Galactolite assay kit (Tropix). All assays were completed on at least three separate occasions. P-values were calculated using a Student’s two-tailed *t*-test.

### 4.4. Yeast Two-Hybrid Assay

A yeast two-hybrid assay was performed by Dualsystems Biotech AG, Zurich, Switzerland using pLexA-DIR-metadherin-aa73-582 as bait and a human placental cDNA library as described [5]. 

### 4.5. Cell lysates, Western Blotting and Immunoprecipitation

All Western blotting procedures were performed as described [49]. Membranes were incubated with primary antibody; anti-metadherin antibodies AK (recognises residues ^197^SHREKRQQRKRDKV^210^) and SS (recognises ^568^SPKQIKKKKKARRET^582^), 1:2000 supplied by Heidi Sutherland (MRC Human Genetics Unit, UK) [45], anti-YTHDC1 1:3000 supplied by Stefan Stamm [17], anti-KHDRBS3 (tSTAR) 1:500 (HPA000275, Sigma Aldrich), anti-Sam68 1:1000 (ab109197, Abcam) and anti-β-actin 1:10,000 (ab6276, Abcam). All secondary antibodies (DakoCytomation) were used at 1:1000. Proteins were detected with ECL-Plus (GE Healthcare) and where they exceeded the dynamic range of film diaminobenzidine (Vector Laboratories, UK) was used. Immunoprecipitation was performed at 4 °C using the method previously described [7]. 

### 4.6. Confocal Microscopy

Cells were grown on glass coverslips in 24-well plates, transfected with 1 µg of DNA per 6 wells of a 24-well plate using FuGENE6. After 48 hours cells were fixed stained and mounted as previously described [7]. Images were taken using a Zeiss Meta 510 confocal microscope using a 63x objective. All scale bars represent 10 µM.

### 4.7. Tissue Microarray and Immunohistochemistry

All immunohistochemistry was performed using a Bondmax Autostainer (Leica) using KHDRBS3 (T-STAR) antibody (1:400, HPA000275, Sigma Aldrich) and citrate buffer pH 6.0 (ER1) for antigen retrieval. The specificity of antibody staining was validated using qPCR and immunohistochemistry of a formalin-fixed paraffin-embedded COS7 cell pellets transiently transfected with siRNA against T-STAR or a non-targeting control (Figure S3, Supplementary Materials). Loss of staining in the specific cellular compartment in up to 50% of cells in the siRNA group alongside a significant reduction in mRNA was considered specific. 

Tissue microarrays constructed from needle biopsies collected from the PICTURE trial consisted of paired tissue from 249 men as previous described [35]. Slides were scanned using a NanoZoomer-SQ digital slide scanner (Hamamatsy Photonics, Japan) and each individual core was assessed for the presence of cancer by a trained uro-pathologist (AF) as well as being scored by two independent scorers (HW and VS) without prior knowledge to clinical data. Cores were scored using the h-score method where an intensity score (0 = none, 1 = weak, 2 = intermediate, 3 = strong) was multiplied by the percentage of epithelial cells with that score; h-score = (% no staining × 0) + (% weak staining × 1) + (% moderate staining × 2) + (% strong staining × 3) [50]. Informed consent was obtained for all patients prior to the creation of the tissue microarray (TMA).

### 4.8. Statistics and Data Analysis

All statistical analysis was performed using either IBM SPSS version 24 software or the R statistical environment. The CD44 exon-specific expression from the TCGA PRAD provisional cohort was downloaded from TSVdb: a web-tool for TCGA splicing variants analysis. Available: http://www.tsvdb.com. This data was matched with clinical data downloaded from the broad firehose, available: https://gdac.broadinstitute.org/. Discretization of expression for survival analysis and survival analysis was performed using the MaxStat and survminer R packages respectively.

## 5. Conclusions

Our results support research that splicing machinery components may become dysregulated in prostate cancer and may do so in response to androgen signaling. They also suggest for the first time, a potentially important role for metadherin, a known oncogene, in regulating alternative splicing in genes with potential clinical significance in prostate cancer. Recent literature has identified metadherin as a potentially valuable therapeutic target, therefore, elucidating key interaction interacting proteins and modulators of metadherin’s function may also have future relevance to targeted therapies.

## Supplementary Materials

The following are available online at https://www.mdpi.com/2072-6694/11/9/1233/s1: Figure S1. YTHDC1 co-localises with the splicing factor SRSF2. Figure S2: Androgen regulation of metadherin, Sam68 and YTHDC1. Figure S3: T-STAR knockdown in COS7 cells. Figure S4: Defining the parameters for the CD44v5-luc assay.
