# Supplementary material for: The Oncogene Metadherin Interacts with the Known Splicing Proteins YTHDC1, Sam68 and T-STAR and Plays a Novel Role in Alternative mRNA Splicing
Source: Cancers (Basel). 2019 Aug 23;11(9):1233. doi: 10.3390/cancers11091233 (PMC6770463; doi:10.3390/cancers11091233)
Supplement: Supplementary file 1 [file cancers-11-01233-s001.pdf]

Figure S1: YTHDC1 co-localises with the splicing factor SRSF2

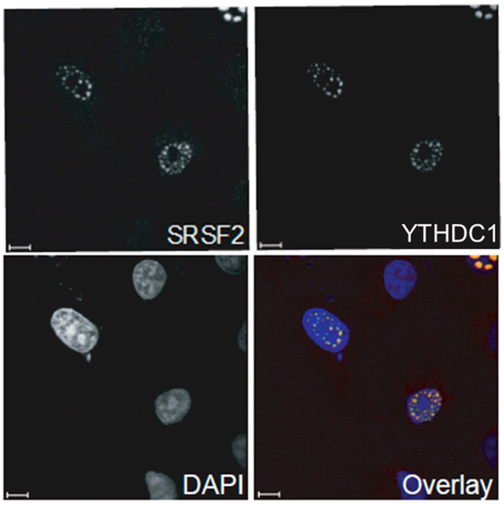

Figure S1. YTHDC1 co-localises with the splicing factor SRSF2. COS7 cells were grown to 70%–80% confluency before being fixed, stained and mounted as previously described 34. Cells were probed with an anti-SC35 (red) and anti-YT511 (green) antibodies then mounted with 46-diamidino-2-phenylindole (DAPI) (blue). Images were taken using a Zeiss Meta 510 confocal microscope using a 63× objective. All scale bars represent 10 µM.

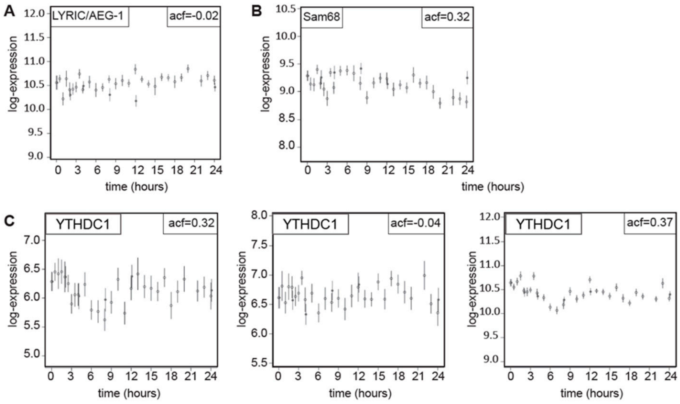

Figure S2. Androgen regulation of metadherin, Sam68 and YTHDC1. LNCaP cells were treated with the synthetic androgen R1881 and RNA samples were extracted at the time points shown 22. Metadherin (A) and Sam68 (B) were detected with a single probe each. YT521 (C) was detected with three independent probes. R1881 treated (open circles), vehicle treated controls (closed circles).

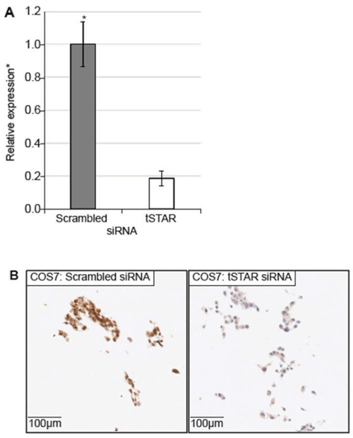

Figure S3. T-STAR knockdown in COS7 cells The specificity of antibody staining was validated using immunohistochemistry of a formalin-fixed paraffin-embedded COS7 cell pellets transiently transfected with siRNA against T-STAR or a non-targeting control. SMARTpool ON-TARGETplus KHDRBS3 (T-STAR) siRNA was purchased from Dharmacon, USA. siRNA (600pM per 10cm dish) was transfected using Lipofectamine (Life Technologies) following manufacturers protocol. Cells were grown a further 72 hours before quantitative polymerase chain reaction (qPCR) and immunohistochemical analysis. Loss of staining in the specific cellular compartment in the siRNA group alongside a significant reduction in mRNA confirmed by qPCR was considered specific. (A) T-STAR expression was determined using qPCR. (B) Cell pellets of transfected COS7 cells were formalin fixed, paraffin embedded and stained for T-STAR to assess protein expression.

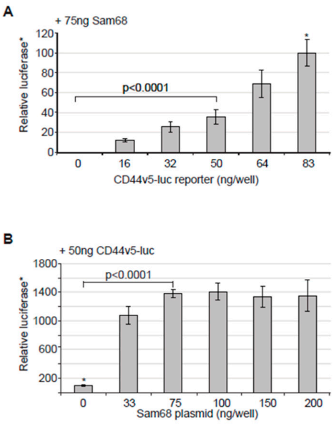

Figure S4. Defining the parameters for the CD44v5-luc assay**.** (A) The CD44v5-minigene plasmid was transfected into COS-7 cells in increasing concentrations alongside a BOS β-galactosidase plasmid to control for transfection efficiency. The empty pSG5 vector was used to equalise the amount of DNA transfected. After 48 hours cells were harvested on ice for 15 minutes using reporter lysis buffer. Luciferase activity was measured using the Luclite luciferase assay kit and results were normalised using a β-galactosidase activity. (B) Using 50ng/well of CD44v5-luc increasing concentrations of SAM68 were transfected into COS-7 cells with a β-galactosidase plasmid. Cells were harvested and

Commented [G1]: Please define if appropriate.

assayed as for (A). All data were combined from at least n = 3. P-values were calculated using a two-tailed *t*-test.
